# Structure and Mechanical Response of Polybutylcarbosilane Dendrimers Confined in a Flat Slit: Effect of Molecular Architecture and Generation Number

**DOI:** 10.3390/polym15204040

**Published:** 2023-10-10

**Authors:** Andrey O. Kurbatov, Nikolay K. Balabaev, Kirill A. Litvin, Elena Yu. Kramarenko

**Affiliations:** 1 Faculty of Physics, Lomonosov Moscow State University, Moscow 119991, Russia; kurbatov@polly.phys.msu.ru (A.O.K.); litvin@polly.phys.msu.ru (K.A.L.); 2 Enikolopov Institute of Synthetic Polymeric Materials RAS, Moscow 119991, Russia; 3 Institute of Mathematical Problems of Biology RAS—The Branch of Keldysh Institute of Applied Mathematics of Russian Academy of Sciences, Pushchino 142290, Moscow Region, Russia; balabaevnk@gmail.com

**Keywords:** dendrimer, polybutylcarbosilane dendrimer, molecular dynamics simulations

## Abstract

Due to the absence of specific interactions, carbosilane dendrimers are ideal models to study the effect of a hyperbranched regular structure on the molecular response to external influences. In this work, we have studied the conformational behavior of single polybutylcarbosilane dendrimers under confinement between impermeable flat surfaces using atomistic molecular dynamics simulations. Dendrimers of different generations belonging to two homologous series with a tetra-functional core and three- and four-functional branches were simulated. The analysis of the dependence of the internal energy of the dendrimers on the wall distance allowed us to determine the critical degree of compression at which the dendrimers are able to change their shape without energy loss. The effects of generation number and branching functionality on the number of wall contacts, density distribution and shape changes were elucidated. It was found that for high generation dendrimers, the inner layers are not accessible for external interaction. It was shown that the excess stresses occurring at high compressions are concentrated in the structural center of the dendrimer. The nature of the elastic response, which is strongly nonlinear, was analyzed at different compressions depending on the dendrimer architecture and generation. We believe that our results are useful for further studies of dendrimer films under compression and can also serve as a basis for developing model concepts to describe the dynamics of dendrimer melts.

## 1. Introduction

Dendrimers are hyperbranched synthetic polymers with a regular tree-like structure. The structural parameters of dendrimers are (i) the functionality of the core, i.e., the number of chains branching from the central atom or group of atoms, (ii) the length of the chains (spacers) with multifunctional end groups (branching points), (iii) the functionality of the branching points, and (iv) the generation, i.e., the number of branching layers [1,2]. Due to their specific architecture, these molecules offer a unique combination of monodispersity and high functionality, which are of particular interest for their practical applications as nanocontainers for targeted drug and gene delivery in medicine [3,4,5,6,7,8] and as templates for various methods of polymer characterization [9]. In addition, the practical potential of dendrimers has been greatly enhanced by the ongoing development of synthetic strategies to increase the variety of chemical compositions and structures [10]. In particular, dendrimers are now widely used to create nanoscale hybrid materials with unique electronic/optical/magnetic properties [11,12,13].

Despite a significant amount of work in the field of dendrimers, the fundamental understanding of the structure–property relationships of these highly branched macromolecules is not yet complete, but is crucial for the further development of new materials based on them. So far, the main practical applications have been at the level of individual macromolecules, mainly of low generations, which have the advantages of monodispersity and a tree architecture and, at the same time, do not require laborious and expensive synthesis. Thus, single dendrimer behavior in dilute solutions has been the focus of much theoretical, computer simulation and experimental research, and is now fairly well-understood [14,15]. Not much research has explored the effect of the dendrimer tree-like architecture on the collective properties of dendrimer systems, such as films, concentrated solutions and melts, where dendrimer molecules strongly interact with each other. The experimental results on the melt rheology were reported for polyether [16], poly(propyleneimine) [17], polyamidoamine [18,19] and polybuthylcarbosilane [20] dendrimers. Computer simulation studies within coarse-grained approaches were aimed at a description of basic thermodynamic characteristics of dendrimer melts [21,22,23,24].

Among a wide variety of chemical compositions of dendrimers, it is worth noting, especially for the class of dendrimers, in which branching occurs due to valence in silicon atoms, namely, carbosilane and siloxane dendrimers [25,26,27], that these dendrimers represent good model systems to study pure effect of tree-like architectures, since they do not contain any groups with specific interactions like, for instance, hydrogen bonding or electrostatic interactions, which contribute a lot into the intermolecular interactions. To date, a large amount of experimental material has been collected on the behavior of polybutylcarbosilane dendrimers in bulk, depending on core and branching functionalities [20,26,28,29,30,31,32,33,34,35,36,37]. Two fascinating phenomena observed experimentally should be highlighted. First, melts of low-generation dendrimers are liquids, while melts of high-generation dendrimers behave more like solids. The liquid-to-solid transition is realized within one generation step. The “transition” generation depends on the structural parameters of the dendrimer. It was shown that the transition for the homologous series with tetra-functional core and three-functional branching Si-atoms takes place between the 5th and 6th generations and is accompanied by an enormous (six orders of magnitude) growth of the melt viscosity [20]. Second, a melt of high generations, with a sufficiently accurate preparation, can represent a solid with a well-defined structure; that is, the periodic spatial arrangement of the dendrimers, in particular, crystallization of the G6 dendrimer melt was reported in Ref. [38]. Recent atomistic simulations of carbosilane dendrimer melts [35,36,37] failed to unequivocally identify the physical mechanism of such peculiar behavior of high-generation dendrimer melts due to long relaxation times and the necessity to model big systems, which are time consuming. Simulation studies of the shear-stress relaxation of low-generation functionalized carbosilane dendrimers, i.e., dendrimers with additional branching segments in their structure, have shown the prevalence of the melt elastic response over the viscous one, in which some frequency range of external load and the authors hypnotized that this phenomenon can be caused by a crowded environment due to internal dendrimer densification [39].

The purpose of this work is to study the elastic response of an individual polybutylcarbosilane dendrimer in dependence of its architecture and generation using atomistic molecular dynamics simulations. To the best of our knowledge, the conformational behavior of these dendrimers under confinement has not been consistently studied, yet. One can mention only one work [40] that touched this question for some carbosilane dendrimers without any comparative analysis; moreover, the research methodology was different; see the next section. On one hand, since elasticity of an individual molecule controls its many-body behavior, and the study of the single molecule mechanical response can help to elucidate the role of deformation in the dynamics of dendrimer melts and create the basis for the development of a simplified model of a melt. On the other hand, it gives some insight into the tree-like macromolecule behavior in AFM research [41,42,43,44]. Such studies make it possible to determine the shape and packing of adsorbed dendrimers but fail in connecting them with the internal structure of these molecules. Thus, the results of our simulations can serve as the basis for further studying films and adsorbed layers of dendrimers.

In the next section, we describe the research objects and simulation methodology. We start the discussion of the results with the analysis of the potential energy of dendrimers under confinement and determine the range of deformations that do not cause any significant strains in the dendrimer internal structure, and gain some insight in the distribution of strains in highly deformed states. Then, we discuss the stress–strain curves, strain-stiffening effects and the influence of the dendrimer structure and generation on the effective elastic coefficients. The further analysis of the number of contacts of various structural elements of dendrimer molecules with the walls allows us to determine the availability of the dendrimer interior for interactions. The shape changes of the whole dendrimer and its interior and peripheral layers show the conformational variations depending on generation and strain degree. Finally, we calculate the linear and volume density profiles and formulate some conclusions.

## 2. Research Objects and Method of Simulation

**Figure 1:**
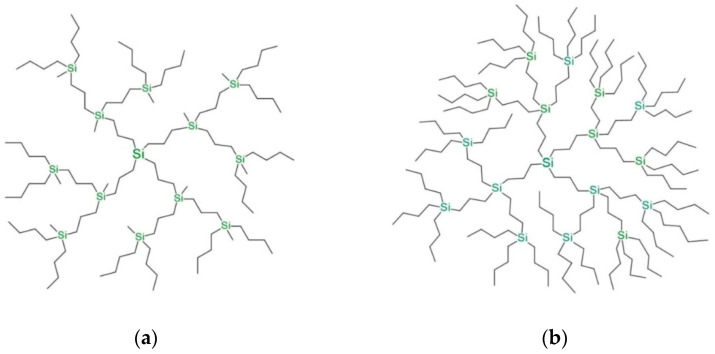
Schematic representation of the second generation polybutylcarbosilane dendrimers of two homologous series (**a**) 4-3G2 and (**b**) 4-4G2.

Two homologous series of poly(butylcarbosilane) dendrimers with a tetrafunctional =Si= core but different branch point functionalities (trifunctional ≡Si(CH_3_) and tetrafunctional =Si=) have been studied (Figure 1); we refer to these series as 4-3 and 4-4 series. The spacers consist of three methylene groups -(CH_2_)_3_- for both homologous series. The terminal layers of dendrimers 4-3 and 4-4 are also the same, consisting of two butyl -(CH_2_)_3_-CH_3_ groups and one methyl -CH_3_ group for each Si atom. As in our previous simulations [45,46], methyl and methylene groups were treated as united atoms. The 4th to 7th and 3rd to 5th generation dendrimers were modelled for the 4-3 and 4-4 homologous series, respectively. It should be noted that due to the different functionalities of the branching points, the number of atoms in dendrimers of the same generation, but belonging to different homologous series, differs dramatically. The total number of atoms, N, in each dendrimer studied is given in Table 1.

The AMBER force field was used to simulate dendrimers, with potentials accounting for bond stretching, bond bending and dihedral angle rotation around equilibrium values. Non-bonded interactions were described by the 12-6 Lennard-Jones (L-J) potential with a cut-off distance of 1.05 nm. Electrostatic interactions due to partial charges on Si atoms and methylene groups were also taken into account. The detailed description of the preparation of the initial non-overlapping dendrimer conformations as well as the relaxation of the dendrimer is given in our previous work in this field [37,45,46]. In all simulations the temperature was fixed at 350 K.

All simulations were carried out using the GROMACS 2020.1 software package [47,48,49,50]. Initially, a single dendrimer was placed in a fairly large rectangular modelling cell. Along the Z-axis, the initial distance from the atoms at the periphery of the dendrimer to the cell boundary was 0.2 nm; along the other two axes, it was equal to at least one size of the dendrimer. Periodic boundary conditions were applied in the X and Y directions, while the cell boundaries along the Z axis were represented by impenetrable walls. Interactions of all dendrimer atoms, regardless of their chemical nature, with the walls, were described by a 9-3 LJ potential, with a parameterization corresponding to methyl particles and a number density of 1 nm^−3^.

**Figure 2:**
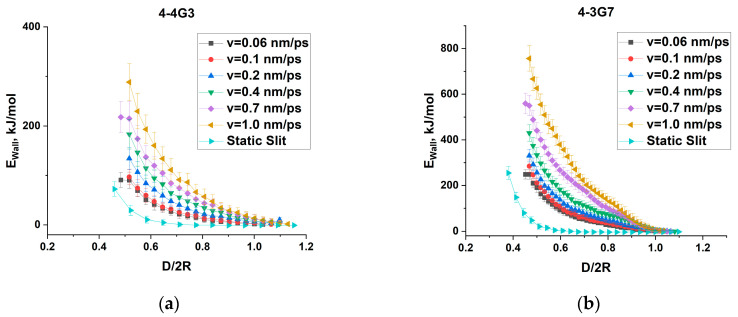
Interaction energy with the walls for (**a**) 4-4G3 and (**b**) 4-3G7 dendrimers as a function of the normalized distance between the walls, D/2R, for different compression rates and for slits of static size as indicated in the legend. 2R = 2R_g_ × (5/3)^0.5^, where R_g_ is the radius of gyration of the dendrimer in the initial uncompressed state.

**Figure 3:**
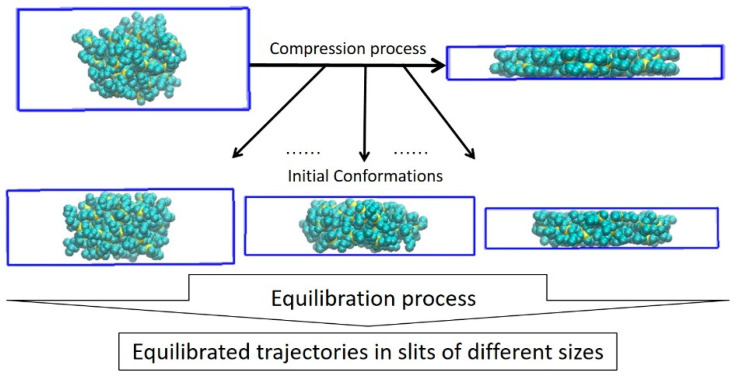
Study design.

The cell containing a dendrimer was then deformed along the vertical Z-axis, i.e., the distance between the impenetrable walls was gradually reduced, resulting in compression of the dendrimer in one dimension. Following the methodology developed in [40], the compression rate was first varied from 1.0 nm/ps to 0.06 nm/ps and the energy E_wall_ of dendrimer interaction with the walls was calculated as a function of wall distance D at each compression rate (Figure 2 and Figure S1). The dependences E_wall_ (D) appeared to be strongly rate-dependent, although there was an apparent tendency towards saturation. To avoid any uncertainty and to obtain equilibrium dendrimer conformations at each D, the following algorithm was applied. The compression rate was further reduced to 0.002 nm/ps and the resulting conformations of the compressed molecule at different distances between the walls were further equilibrated for 100 ns each. All of the characteristics presented below were obtained by averaging the last 50 ns of the trajectories. The design of this study is shown schematically in Figure 3. One can see in Figure 2 that dendrimer relaxation causes conformational changes which resulted in a considerable reduction in E_wall_ that is especially pronounced for high-generation dendrimers.

The adopted procedure ensures that the conformations of the dendrimers are in equilibrium at each degree of compression and allows one to eliminate any effects of the compression rate as well as various additional factors, such as the presence or absence of temperature control during compression [40]. Based on the analysis of various dendrimer characteristics, such as the time dependence of the potential energy contributions, and the shape and size of the dendrimers, it can be argued that the obtained trajectories are well-balanced and the results of their analysis are correct. Such a simulation gives a more accurate idea of the behavior of a real dendrimer, especially in an AFM study, since experimental compression rates are orders of magnitude lower than those available in computer simulations.

## 3. Results and Discussion

### 3.1. Potential Energy

**Figure 4:**
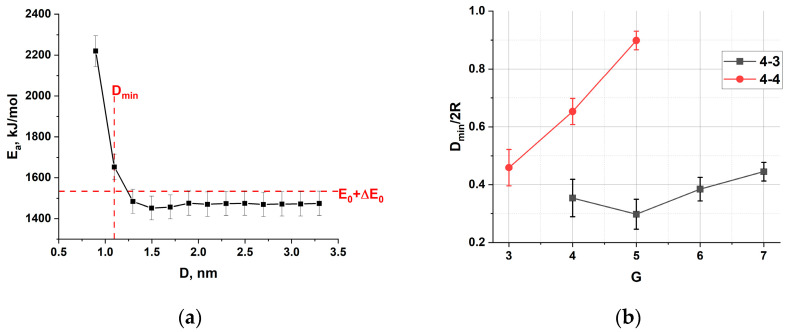
(**a**) Dependence of the angle energy on the wall distance for a 4-3G4 dendrimer. The red line shows how the critical distance D_min_ is calculated. (**b**) D_min_ versus generation number G for all dendrimer types investigated. The values are normalized to 2R = 2R_g_ × (5/3)^0.5^, where R_g_ is the radius of gyration of the dendrimer in the initial uncompressed state.

Reducing the distance between the walls below the average size of the dendrimer obviously leads to a change in the dendrimer conformations. First, we investigate how this change affects the potential energy contributions due to bonds, valence and torsional angles. We have calculated the dependence of these contributions on the distance between the walls for all the dendrimers studied. Their behavior, characterized by a constant value for large D and a sharp increase below a certain critical D, is common for all potential energy contributions and for all types and generations of dendrimers studied. As an example, Figure 4a shows a typical dependence for the valence angle energy of the 4-3G4 dendrimer. It should be noted that for the AMBER force field, where the potentials are represented in a harmonic form, a large deviation of the potential energy from the equilibrium value indicates the inapplicability of the model. In fact, the increase in any potential energy contribution corresponds to the occurrence of some stresses in the dendrimer structure, i.e., deformations of bonds and angles in comparison with their values for uncompressed dendrimers.

To estimate the critical compression of the dendrimer, we found the distance between the walls, D_min_, at which the average value of at least one potential energy contribution exceeds the average equilibrium value E_0_, taking into account the error ΔE_0_, i.e., the value of E_0_ + ΔE_0_ (see Figure 4a). This critical distance D_min_ depends on the architecture of the dendrimer. The dependence of the normalized D_min_ on the dendrimer generation for the two homologous series is shown in Figure 4b. D_min_ was normalized to 2R = 2R_g_ × (5/3)^0.5^, where R_g_ is the radius of gyration of the dendrimer in the initial uncompressed state.

It can be seen that the dendrimers of the homologous series 4-4 have a rather rigid structure. The value of D_min_ increases monotonically with the generation number. Furthermore, the critical relative deformations of the dendrimers are quite low; in particular, it is sufficient to compress the 4-4G5 dendrimer by only 10% to cause deformations of the intramolecular bonds. The dendrimers of the homologous series 4-3 are more deformable. This difference can be explained by a denser molecular structure of the homologous 4-4 series due to a higher branching functionality. In addition, this molecular density of the 4-4 series grows faster with generation (see Table 1). As a result, the repulsive part of the van der Waals interaction begins to play a role at smaller relative compressions of the 4-4 dendrimers, and the total energy is minimized by redistribution of potential energy contributions (bonded vs. unbonded) leading to structural deformations.

**Figure 5:**
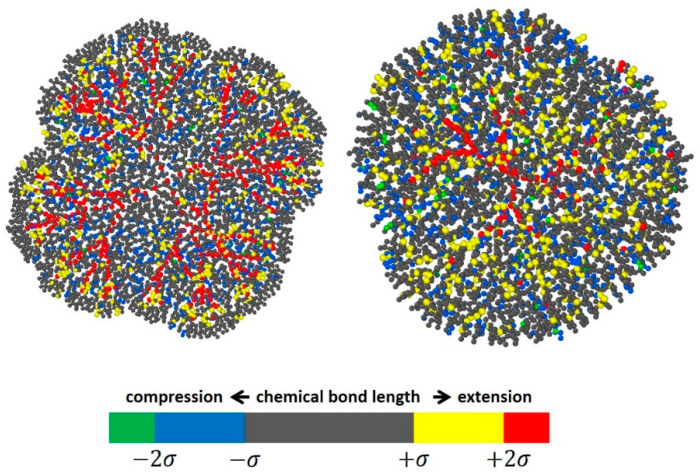
Snapshots of the 4-4G5 (**left**) and 4-3G7 (**right**) dendrimer in the highly deformed state at D/2R = 0.38. Different colors show the degree of deformation according to the distribution of the bond length (Figure S2), which is approximated by the Gaussian distribution with standard deviation σ.

Although formally the model fails in simulations of dendrimer conformations at too high compressions, when too much stress appears in chemical bonds and valence angles, it would be interesting to see in which structural part of the dendrimers the stress occurs. Figure 5 shows the frontal projection of the 4-4G5 dendrimer at D/2R = 0.38. Different colors indicate different bond lengths, i.e., the deviation from the potential minimum. It can be seen that the excess stress is mainly concentrated in the structural center of the dendrimer, which is one of the properties inherent to this type of compound. A similar effect has been observed for high generations of siloxane dendrimers with extremely short spacers consisting of a single oxygen atom, for which stresses appear in individual dendrimers without any confinement, simply because the structure is too dense [51]. The presence of spatial confinement enhances this effect, which begins to appear for dendrimers with sufficiently long spacers that are unstressed in the free state of the dendrimer.

### 3.2. Dendrimer Elasticity

**Figure 6:**
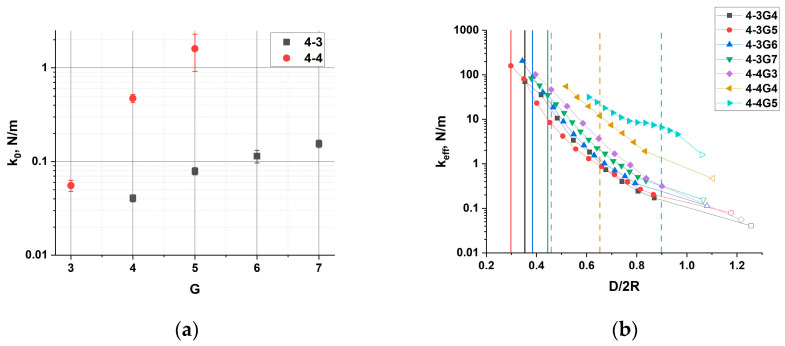
(**a**) Dependence of the effective elastic coefficient k_0_ in the limit of small deformations on the dendrimer generation. (**b**) Dependence of the effective elastic coefficient on the relative distance D/2R between the walls, 2R = 2R_g_ × (5/3)^0.5^, where R_g_ is the radius of gyration of the dendrimer in the initial uncompressed state. Vertical lines show the critical distance, D_min_/2R, at which strains of internal structure appear. Colors correspond to the legend. The empty symbols correspond to the k_0_ values of the corresponding dendrimers shown in (**a**).

To characterize the mechanical response of the dendrimer to compression, we calculated the interaction force F of each dendrimer with the walls as a function of the distance D between them. The force was obtained from the pressure tensor. Functions F(D) for all dendrimers studied are shown in Figure S3. Fitting the initial part of the curve with a linear dependence (see the insets of Figure S3), we obtain an effective elastic coefficient k_0_ of the dendrimer, which characterizes its elastic response at small deformations (Figure 6a).

Figure 6a shows that the effective elastic coefficient grows with the generation number for both homologous series, but the absolute value of k_0_ and its relative change with generation are markedly different for the 4-3 and 4-4 dendrimers. For the 4-4 series, more than an order of magnitude increase in k_0_ is observed as the generation increases from 3 to 5. For the 4-3 series, the elastic coefficient of the G7 dendrimer is only about 4 times that of the G4 dendrimer. In addition, the entire 4-3 series is significantly softer than even the G4 dendrimer of the 4-4 series. Thus, the functionality of the branching point plays a crucial role in the mechanical response of dendrimers.

It should be noted that the F(D) dependences are strongly non-linear. To characterize this non-linearity, we define the effective tangential elastic coefficient, k_eff_, as the slope of the F(D) curves at different D. The value of k_eff_ at each D is calculated as the tree-point average derivative of F(D). The dependences of k_eff_ on D are shown in Figure 6b for all dendrimers studies. The empty symbols correspond to the values of k_0_ realized at small deformations in the linear regime (Figure 6a). For 4-3 series, one can see an almost three-order increase in the value of k_eff_ with decreasing D, i.e., with increasing deformation, before D_min_ is reached. This tremendous stress-stiffening effect is observed for dendrimers of all generations studied and can be attributed to their highly branched structure decreasing their conformational freedom in comparison with linear macromolecules.

### 3.3. Interactions with the Walls

**Figure 7:**
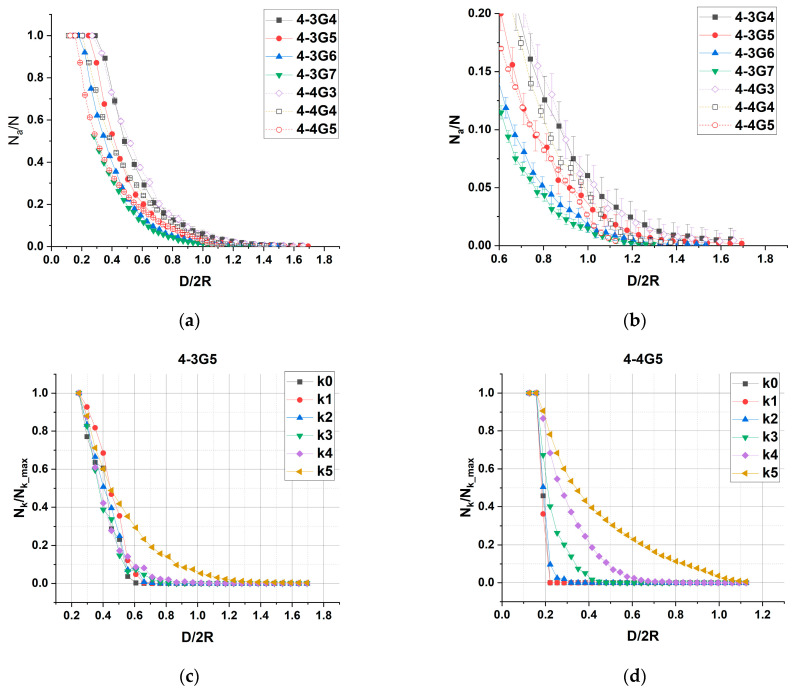
(**a**) Dependence of the relative number, N_a_/N, of dendrimer atoms in contact with the walls on the relative wall distance D/2R and (**b**) the same magnified for large wall distances for all dendrimers studied. (**c**,**d**) Dependence of the relative number, N_k_/N_k_max_, of dendrimer atoms belonging to the k-layer in contact with the walls on the relative wall distance D/2R for the 4-3G5 and 4-4G5 dendrimers, respectively. N_k_max_ is the maximum number of atoms in the k-layer.

Figure 7 shows the dependences of the relative number N_a_/N of dendrimer atoms in contact with the walls on the wall distance. The value of N_a_ is calculated as the number of atoms in a thin layer Δz = 0.5 nm near the walls. While the absolute number of surface contacts increases in proportion to the total number of atoms in the dendrimer (Figure S4), the relative number of contacts behaves in a more complex way. For wall separations larger than the dendrimer diameter, the value of N_a_/N tends to zero for more rigid dendrimers, while it takes non-zero values for more deformable dendrimers, these being dendrimers of small generations of both homologous series (4-3G4, 4-3G5, 4-4G3). At intermediate separation, when dendrimer compression occurs, the smallest number of surface contacts is realized for the high-generation dendrimers of the 4-3 series, presumably due to their less dense structure, which can be deformed more easily than that of the larger and stiffer 4-4G4 and 4-4G5. Overall, the relative number of contacts grows faster with dendrimer compression for the 4-3 series than for the 4-4 series. At high compression N_a_/N tends to unity, in this regime, stresses appear in the dendrimer structure, i.e., in bonds and valence angles.

To study the accessibility of the inner layers of the dendrimers for interaction with the walls as a function of D, we calculate the relative number of contacts for each branching layer separately. At low compressions, only the peripheral layer of dendrimers interacts with the surface for all dendrimers under study. For low-generation dendrimers, the inner layers begin to interact with the surface almost simultaneously at a certain critical compression (Figure 7c). In contrast, for high-generation dendrimers and especially for the 4-4 series, the confining surface is inaccessible to the inner part of the dendrimers even at rather high compressions. A separate successive “punching” of the dendrimer layers with decreasing D can be observed, i.e., intervals of D values where only one layer, or only two layers, etc. is available for interaction with the surface, are quite significant and well separated from each other (Figure 7d). Furthermore, for low-generation dendrimers (G4 and G5 of the 4-3 series and 4-4G3), the atoms of almost all branching layers are available for interaction with the surface before critical compressions are reached. The number of core layers unavailable to wall interaction increases with generation for 4-3 series (these are two layers for 4-3G6 and four layers for 4-3G7), while 4-4G4 and 4-4G5 interact with the walls only through the peripheral layer, without any stress on the internal structure (Figure 7d and Figure S5).

In Ref. [37], we performed the comparative analysis of the structure of 4-3 dendrimer melt as a function of generation by atomistic molecular dynamics simulations. We calculated the density distribution profiles and separated two regions in the dendrimer, namely, the peripheral one, which is penetrated by neighboring molecules, and the internal one, which is inaccessible to outsiders. The radius of the internal impermeable region increases significantly with dendrimer generation (Table S1). While G3-G5 dendrimers can interpenetrate almost the entire molecule, the radius of the inner region of G6 and G7 correlates with D_min_ being slightly larger than D_min_.

### 3.4. Dendrimer Shape

**Figure 8:**
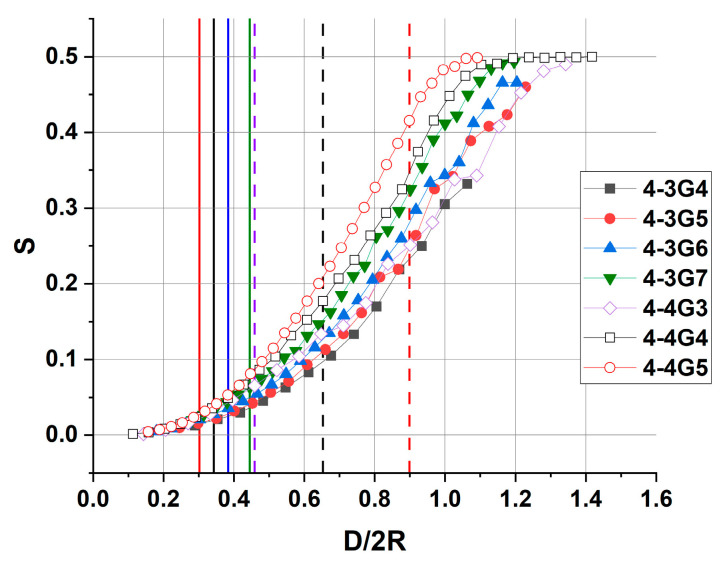
Dependence of the shape factor (*S*) on the relative wall distance D/2R. 2R = 2R_g_ × (5/3)^0.5^, where R_g_ is the radius of gyration of an uncompressed dendrimer. Vertical solid (for 4-3 series) and dashed (for 4-4 series) lines show the value of the critical distance D_min_ for each dendrimer type, colors correspond to the legend.

To assess the change in the shape of dendrimers as a function of the slit size, the parameter $S=\frac{R_{z}^{2}}{R_{x}^{2}+R_{y}^{2}}$ was calculated, where R_x_, R_y_ and R_z_ are the corresponding components of the gyration tensor. For a symmetrical body, such as a sphere, *S =* 0.5. A decrease in this parameter means that a molecule adopts more disc-like conformations. For all the dendrimers studied, the dependence of the shape factor on the relative distance between the walls is shown in Figure 8.

It can be seen that noticeable deformations of the dendrimers occur even in slits of rather large sizes, i.e., the value S becomes smaller than 0.5 at wall distances larger than the effective size of uncompressed dendrimers, D/2R > 1. Deformations at D/2R > 1 are particularly noticeable for the dendrimers of the soft homologous series 4-3. Despite the fact that at D/2R > 1 the dendrimer does not touch both walls at the same time, it hits one or the other during the movement, leading to its flattening. This effect is also observed for linear polymers under conditions of external constraint [51]. As the stiffness of the dendrimer increases, this effect becomes less pronounced, as for example in 4-4G4 and 4-4G5. In general, we see a monotonic flattening of the dendrimers with decreasing slit size, which is qualitatively the same for all dendrimer types. For a given D < 2R, the value of S correlates well with the stiffness of the dendrimer. Namely, the lower the value of the effective elastic constant, the lower the value of S, i.e., softer dendrimers spread more over the surface when compressed.

**Figure 9:**
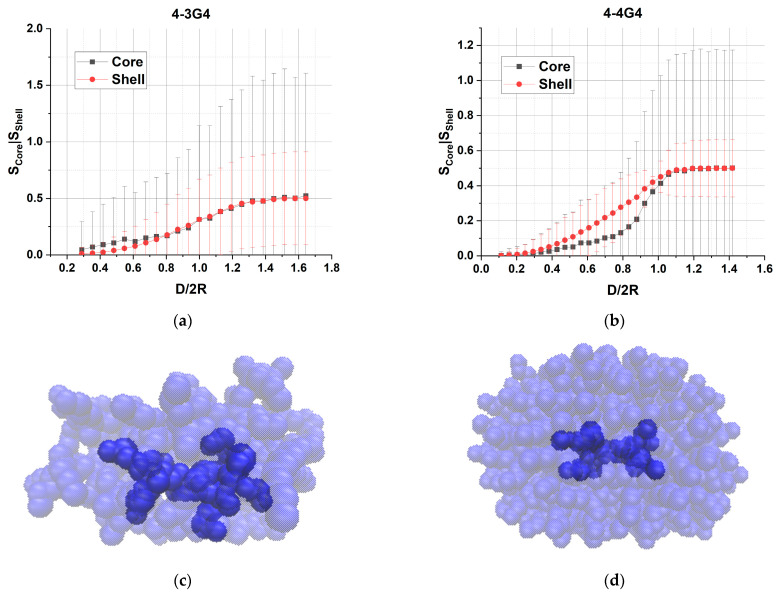
Dependence of the shape factors (*S*) calculated for the inner and peripheral layers of the dendrimers 4-3G4 (**a**) and 4-4G4 (**b**) on the relative distance between the impenetrable walls. The abscissa axis is normalized to 2R = 2R_g_ × (5/3)^0.5^, where R_g_ is the radius of gyration of an uncompressed dendrimer. The snapshots of the dendrimers 4-3G4 (**c**) and 4-4G4 (**d**) at D/2R ≈ 0.8 Atoms of the inner layers are shown by dark blue while the atoms of the peripheral layer are colored by light blue.

To study the effect of the compression on the internal structure of the dendrimers, we calculate separately the shape factors for the internal branching layers of the dendrimer, namely, for the layers from 0 to (G-2), and the peripheral layer k(G) (Figure 9). Note that the layer G-1 is not taken into account. It was found that the shape factor of the peripheral layer monotonously decreases with the wall distance D, which coincides with the shape factor behavior of the whole dendrimer and, thus, demonstrating a flattening of the dendrimer as a whole. However, by comparing the S(D) dependences of the inner and peripheral layers, we found that under moderate compressions, the core region of the dendrimer flattens more than the periphery, which is typical of all the dendrimers studied (an example is shown in Figure 9b). Furthermore, for softer dendrimers (G3–G6 of the 4-3 series and G3 of the 4-4 series) an opposite tendency is observed at high compressions, i.e., the peripheral layer appears flatter. The non-monotonic dependence of the shape factor of the core region was observed for the 4-4G3 dendrimer. It should also be noted that the shape fluctuations of the peripheral layer are mostly hindered by interactions with the walls, while the shape of the inner layers fluctuates to a large extent, so that it can adopt both prolate and oblate conformations at low compressions.

### 3.5. Density Profiles

**Figure 10:**
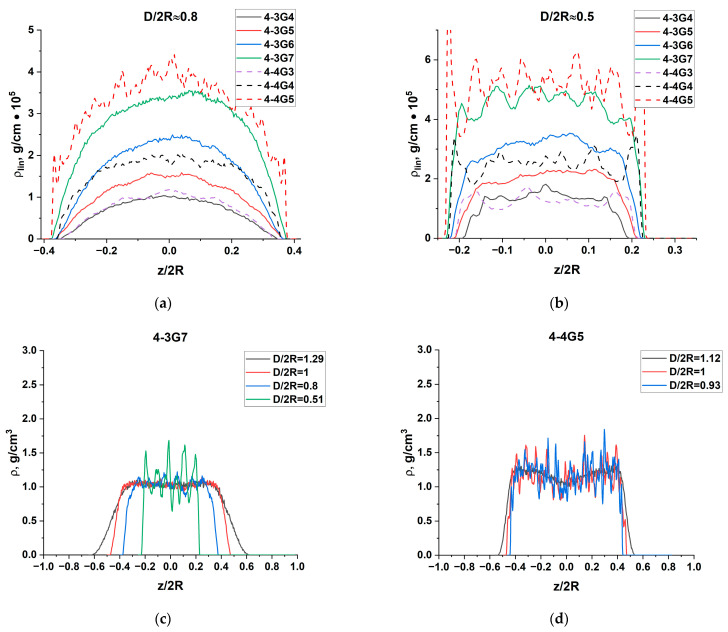
Linear density profiles along the z-axis for all dendrimers studied at D/2R = 0.8 (**a**) and 0.5 (**b**). Density profiles along the z-axis calculated in the cylindrical region of the radius R_g_/2 for 4-3G7 (**c**) and 4-4G5 (**d**) at different D/2R as indicated.

Monomer density profiles along the z-axis were calculated as $\rho_{lin}\left(z\right)=\sum n_{i}\left(z\right)m_{i}/\Delta z$, where $n_{i}$ is the number of atoms of the type *i* in a flat layer [z − 0.5Δz, z + 0.5Δz] of the width Δz parallel to the walls and located at the distance z from the bottom surface, $m_{i}$ is the mass of the *i*-atom, and Δz = 0.2Å. In fact, the calculated value $\rho_{lin}\left(z\right)$ corresponds to the linear density along the z-axis. The functions $\rho_{lin}\left(z\right)$ for D/2R approximately equal to 0.8 and 0.5 are shown in Figure 10a,b for all dendrimers investigated.

It can be seen that for the larger slit D/2R = 0.8, the linear density profile has a bell shape, with a gentle maximum in the center of the slit, and a density decreasing to zero near the walls. The higher the generation, the higher the density. For higher compressions, D/2R = 0.5, the density tends to a constant value almost throughout the slit space, dropping to zero near the walls due to the excluded volume. Density oscillations appearing in the density profile of the 4-3G7 dendrimer and the dendrimers of the 4-4 series are probably related to the intramolecular positions of massive Si branching centers. It should be mentioned that at D/2R = 0.5 the 4-4 series dendrimers experience stresses in bonds and bond angles (see Figure 4b).

To determine whether compression affects the volume density of the dendrimer molecules, we have also calculated the density distribution along the z-axis as the density within the cylinder of the radius R_g_/2, with the axis of the cylinder oriented along the z-axis and passing through the center of mass of the dendrimer, $\rho \left(z\right)=4\sum n_{i}\left(z\right)m_{i}/\left(\pi R^{2}\Delta z\right)$, to exclude the contribution from the lateral unconfined regions. The results for 4-3G7 and 4-4G5 are shown in Figure 10c,d (for the other dendrimers they can be found in SM, Figure S6). It can be seen that the density in the center of the slit remains constant for all compressions. The regions near the slit walls where the density drops to zero become smaller as D decreases.

## 4. Conclusions

Atomistic molecular dynamics simulations of single polybutylcarbosilane dendrimers confined between two impenetrable planar surfaces were carried out. The distance D between the surfaces was varied in a wide range, so that the degree of dendrimer compression varied from light (D/2R~1, where R is the effective radius of a dendrimer) to strong (D/2R << 1). Equilibrium dendrimer conformations were obtained at each D/2R. Dendrimers with the functionality of the central Si atom equal to 4, belonging to two homologous series differing in the functionality of the branched Si atoms (3 and 4), were studied. For the 4-4 series, the dendrimers were simulated from the 3rd to the 5th generation, while for the 4-3 series, the dendrimer generation was varied from the 4th to the 7th generation. The comparative analysis of the dendrimer behavior under compression allowed us to elucidate the effect of the generation number and branching functionality on the elastic response as well as on the internal structure of polybutylcarbosilane dendrimers.

The critical degree of dendrimer compression has been found, above which significant stresses in chemical bonds and valence angles occur. Dendrimers of the 4-3 series can be compressed to 60–70% of their original size in the free state without internal deformation. At the same time, the chemical structure of the denser 4-4 dendrimers is more sensitive to deformation. Even the smallest 3rd generation dendrimer is stressed at less than 60% strain, while the 5th generation dendrimer can only deform 10% without disturbing the bond and valence angle equilibrium.

At small compressions, only the peripheral layer of dendrimers interacts with the confining walls. A decrease in the wall distance causes an increase in the number of contacts of the inner layers of the dendrimers with the confining surfaces. At critical compressions for the 4-3 series, atoms of almost all branching layers interact with the walls. In contrast, the inner layers of the 4-4G4 and 4-4G5 dendrimers are not accessible for external interaction.

The elastic response of all dendrimers investigated is strongly non-linear. It has been shown that a higher density of the molecular structure realized for 4-4 series due to the higher branching functionality results in (i) much larger values of the effective elastic constant, k, of these dendrimers compared to those for the 4-3 series and (ii) a tremendous growth of k with generation. In particular, only a fourfold increase in the effective coefficient is observed from G4 to G7 generations of the 4-3 series. At the same time, the k-value of the 5th generation of 4-4 dendrimers is more than an order of magnitude larger than that of the 3rd generation and it is an order of magnitude larger than the elastic modulus of the largest 4-3G7. A strain-stiffening effect is very pronounced for all dendrimers, it manifests itself in an almost three-orders of magnitude increase in the effective elastic constant with deformation.

It was found that the effect of the confinement on the dendrimer conformations begins to appear at distances between compression planes exceeding the size of the dendrimer. The lower the dendrimer generation, the larger the deviation of the dendrimer shape from the spherical shape at D/2R = 1. At larger compressions, disc-like conformations of dendrimers are realized. The value of the sphericity parameter S correlates well with the dendrimer rigidity, i.e., softer dendrimers spread more over the surface when compressed. The sphericity parameter of the peripheral layer follows the behavior of S for the whole dendrimer while the shape of the core region of the dendrimer fluctuates to a large extent, so that it can adopt both prolate and oblate conformations at small compressions.

It has been shown that the density profile changes from a standard bell-shaped one with a maximum at the center of the slit to the one characterized by an almost constant value decreasing to zero at the walls. It was demonstrated that excess stresses occurring at high compressions are concentrated in the structural center of the dendrimer.

The work carried out helps to deepen the understanding of the behavior of dendrimer molecules under spatial restrictions.

## Figures and Tables

**Table 1:** Number of atoms depending on the type of dendrimer.

Generation	Number of Atoms, N		R_g_, nm	
	4-3 Series	4-4 Series	4-3 Series	4-4 Series
G3		641		1.227 ± 0.010
G4	557	1937	1.201 ± 0.015	1.721 ± 0.005
G5	1133	5825	1.498 ± 0.008	2.403 ± 0.002
G6	2285		1.891 ± 0.008	
G7	4589		2.373 ± 0.007	

## Data Availability

The data presented in this study are available on request from the corresponding author.

## Supplementary Materials

The following are available online at https://www.mdpi.com/article/10.3390/polym15204040/s1, Figure S1: Interaction energy of dendrimers with the walls for different compression rates and for slits of static size, Figure S2: Distribution of bond lengths for 4-4G5 and 4-3G7 for uncompressed conformations and at D/2R = 0.38, Figure S3: Dependence of the force of dendrimer interaction with the walls on the distance between them, Figure S4: The absolute number of surface contacts, Table S1: The size of the internal region of dendrimers, R_core_, inaccessible to neighbors in melts, and its relative values, R_core_/2R, 2R = 2R_g_ × (5/3)^0.5^, Figure S5: Dependence of the relative number of dendrimer atoms belonging to the k-layer in contact with the walls on the relative wall distance, Figure S6: Density profiles along the z-axis calculated in the cylindrical region of the radius R_g_/2 at different D/2R as indicated.
