# Supplementary material for: Structure and Mechanical Response of Polybutylcarbosilane Dendrimers Confined in a Flat Slit: Effect of Molecular Architecture and Generation Number
Source: Polymers (Basel). 2023 Oct 10;15(20):4040. doi: 10.3390/polym15204040 (PMC10610163; doi:10.3390/polym15204040)

polymers

Supplementary Materials

Structure and mechanical response of polybutylcarbosilane dendrimers confined in a flat slit: effect of molecular architecture and generation number.

Andrey O. Kurbatov [1,2], Nikolay K. Balabaev [3], Kirill A. Litvin [1] and Elena Yu. Kramarenko [1,2,*]

[1] Faculty of Physics, Lomonosov Moscow State University, Moscow 119991, Russia; kurbatov@polly.phys.msu.ru

[2] Enikolopov Institute of Synthetic Polymeric Materials RAS, Moscow 119991, Russia

[3] Institute of Mathematical Problems of Biology, Keldysh Institute of Applied Mathematics RAS, Pushchino, Moscow Region 142290, Russia; balabaevnk@gmail.com

* Correspondence: kram@polly.phys.msu.ru; Tel.: +07-495-939-4013

Figure S1. Interaction energy of dendrimers with the walls for different compression rates and for slits of static size. $2R = 2R_g \times (5/3)^{0.5}$, where R_g is the radius of gyration of the dendrimer in the initial uncompressed state.

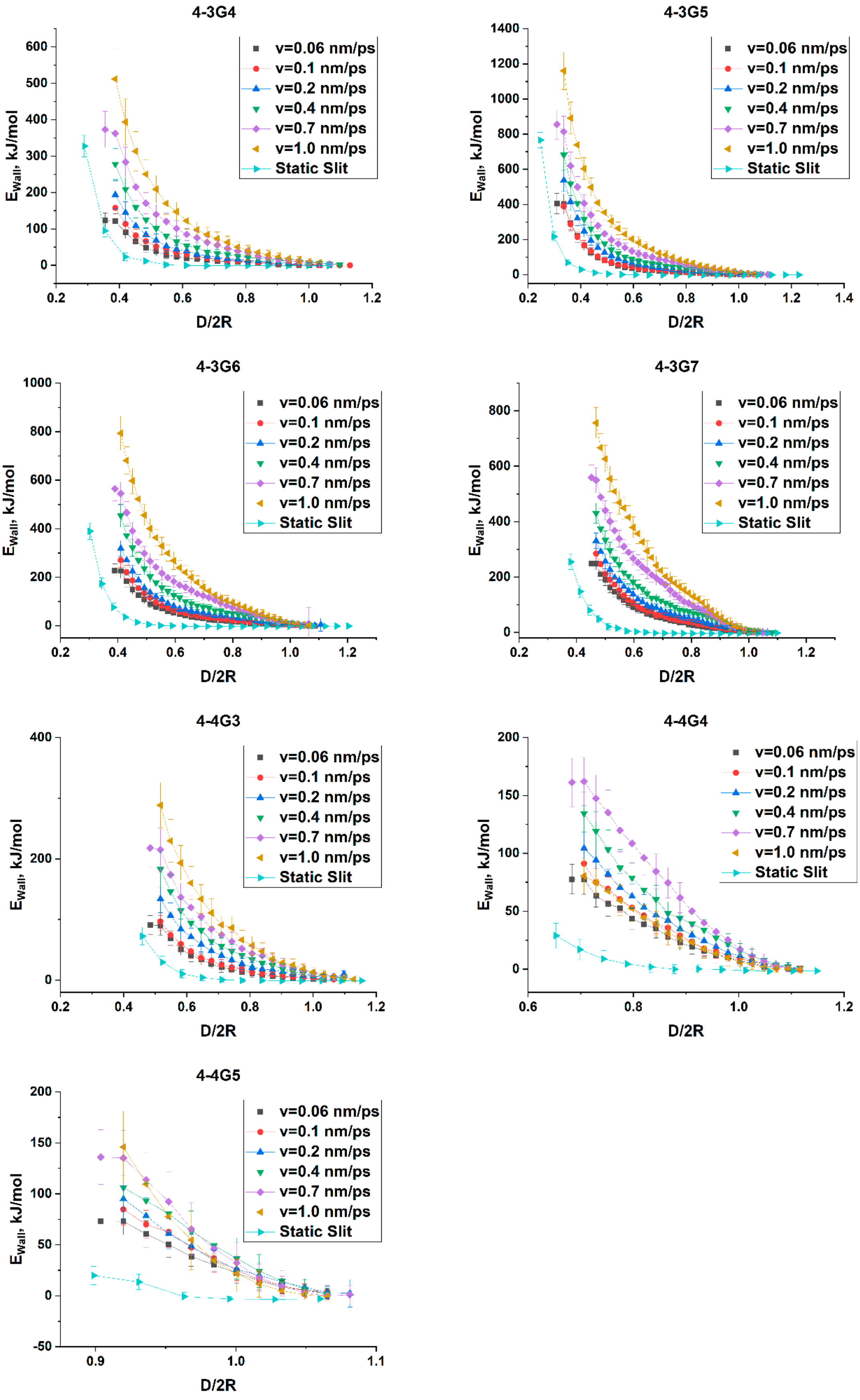

Figure S2. Distribution of bond lengths for 4-4G5 and 4-3G7 for uncompressed conformations and at D/2R=0.38. Bond lengths are normalized to the minimum of the bond potential, and distribution values are normalized to unity. The insets show the tails of the distributions on a larger scale.

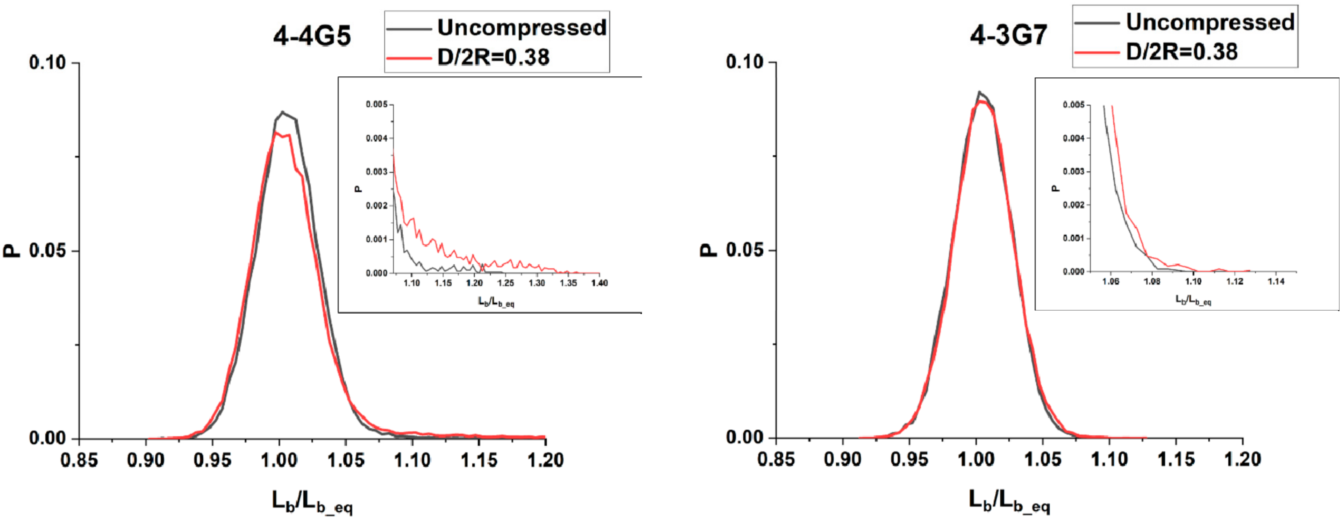

Figure S3. Dependence of the force of interaction with the walls on the distance between them. The inset shows the approximation of the simulation data by a linear dependence in the region of small deformations.

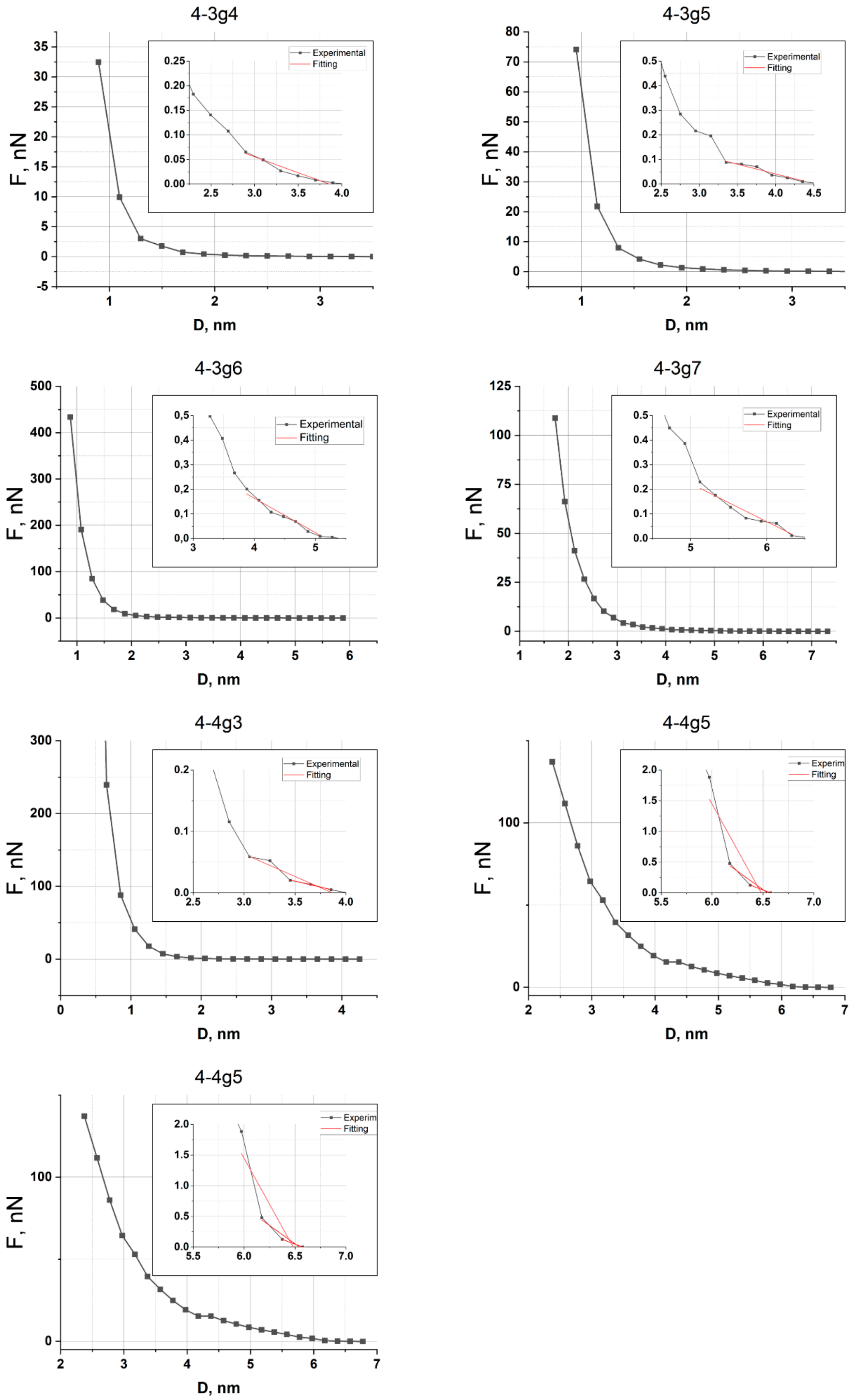

Figure S4. The absolute number of surface contacts.

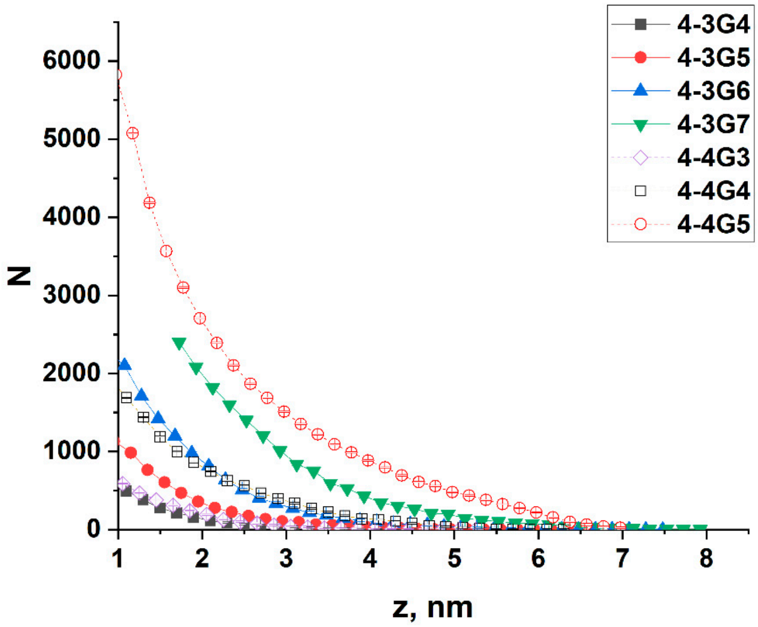

Table S1. The size of the internal region of 4-3 dendrimers, Rcore, inaccessible to neighbors in melts, and its relative values, Rcore/2R, $2R = 2R_g \times (5/3)^{0.5}$. Data were obtained as a result of melt modeling performed in [29].

	Melt Core/2, nm	Melt Core / 2R
4-3G4	0.325	0.210
4-3G5	0.375	0.194
4-3G6	1.025	0.420
4-3G7	1.725	0.563

Figure S5. Dependence of the relative number of dendrimer atoms belonging to the k-layer in contact with the walls on the relative wall distance. Vertical dashed lines show the normalized radius of the internal dendrimer region, $R_{core}/2D$, inaccesible to neighbors in melts.

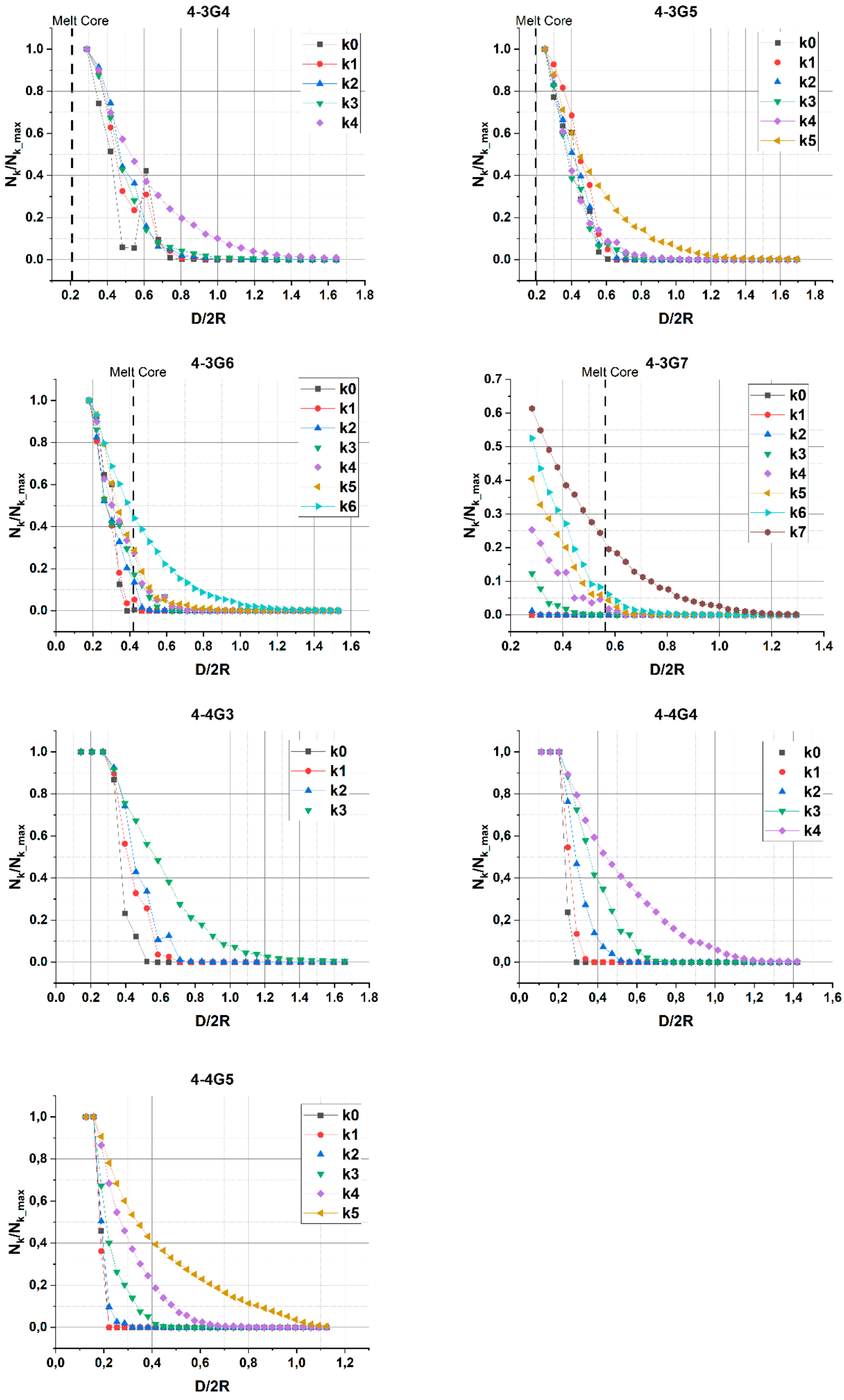

Figure S6. Density profiles along the z-axis calculated in the cylindrical region of the radius $R_g/2$ at different D/2R as indicated.

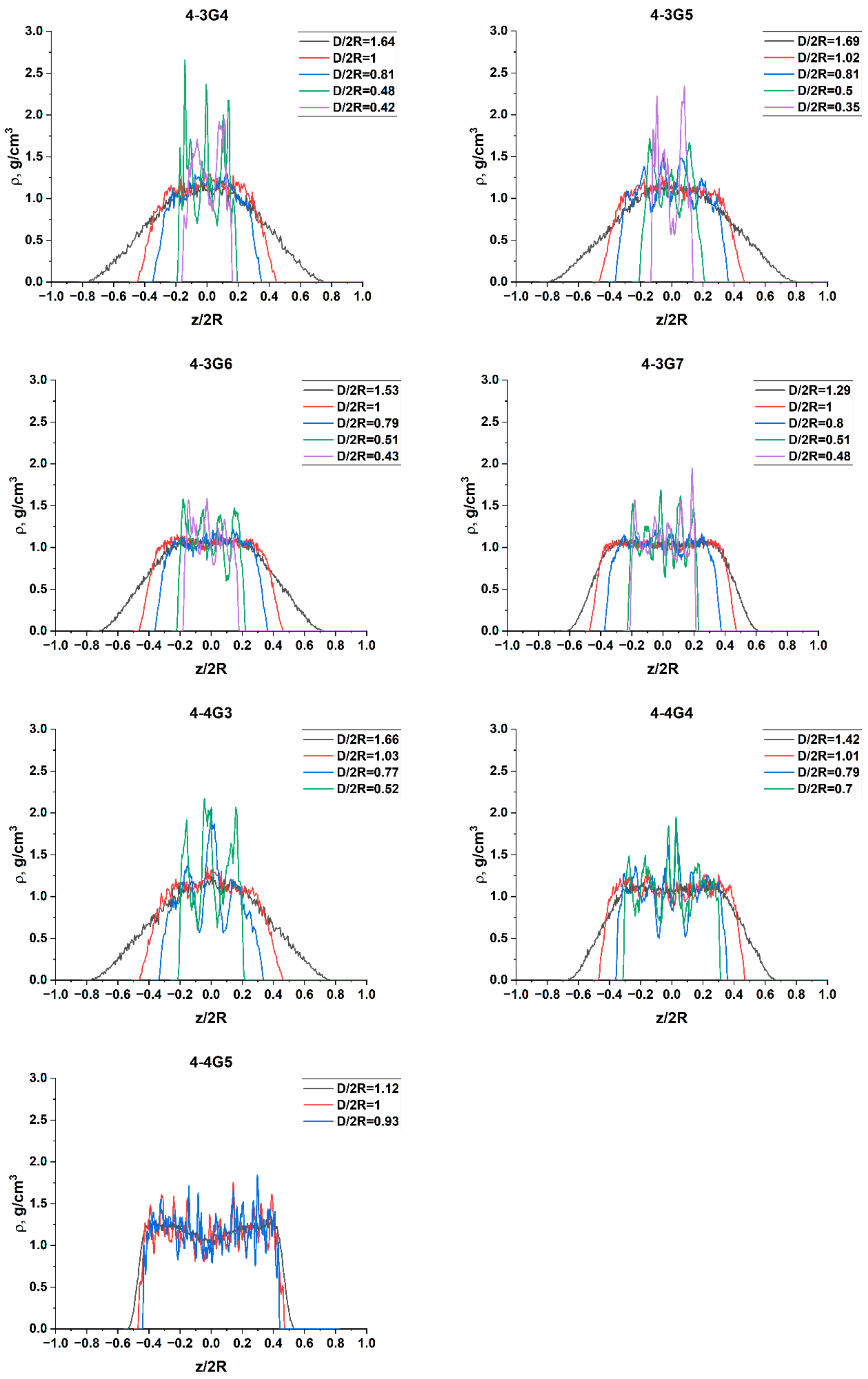

Supplement: Supplementary file 1 [file polymers-15-04040-s001.zip › polymers-2645308-supplementary.pdf]
